# Prostate Cancer and New Insights in Angiogenesis

**DOI:** 10.3389/fonc.2014.00243

**Published:** 2014-09-10

**Authors:** Sanja Štifter, Gordana Đorđević

**Affiliations:** ^1^Department of Pathology, Faculty of Medicine, University of Rijeka, Rijeka, Croatia

**Keywords:** prostate, cancer, angiogenesis

Angiogenesis is considered one of the hallmarks of tumor initiation and progression therefore it is also the widely accepted therapeutic target. It has been shown that angiogenesis blockers prolong patient’s survival for several months (1). In order to progress, cancer cells acquire several distinct morphological alterations increasing their capacity to proliferate and independently sustain growth, and at the same time, inhibit homeostatic, physiologic signals from the microenvironment they are in, and under the control of which they should have remained. This is the main principle behind a tumor gaining the ability to metastasize (2). Till now, several agents have been approved by the Food and Drugs Administration (FDA) but their efficacy is still limited and this observation is repeatedly proven by recurrence of more aggressive tumor after application of anti-angiogenic therapy (3).

So far stated is highly suggestive to back up and justify the existence of hyperplasia in the field of research for novel therapies, which would induce suppression of angiogenesis in carcinogenesis cascade and limit tumor progression. The complexity of angiogenesis becomes one of the greatest challenges in understanding various processes of inflammation, reparation, tumorigenesis, and metastases. In following paragraphs, we will try to express opinion where in near future we should probably search for the next generation of promising therapeutics for management of prostate cancer. It has been established that new blood vessel formation is required for tumor growth (4). The mutations, which predispose tissue to neoangiogenesis and induce tissue invasion, are reactivated genetic programs normally turned off in adult human especially those activated during reparation processes in the surrounding microenvironment, where pro-angiogenic factors play a key role in establishing a capillary network from the surrounding host tissues (5). The surrounding stromal cells, tumor-associated macrophages, and other components of the extracellular also actively constitute matrix chemical milieu, and not just the cancer cells alone as previously implied. To be more precise, angiogenic switch by its definition implies impaired angiogenesis and importance of high vascular endothelial growth factor (VEGF) and VEGF receptor (VEGFR) levels holding responsible for PCA progression (6).

The level of oxygenation in prostate is important for estimation of its hypoxic state. Namely, hypoxic state is informative as relative tissue oxygenation status, which correlates with overall VEGF expression in prostate (7–10). Progressive induction of hypoxia influences metabolic changes at cellular level in prostate epithelial cells and their energetic demands are being modified accordingly. Impaired systems of cellular repair are not capable to restore in full sublethal cellular damage induced by hypoxia therefore pro-inflammatory cytokines are being increasingly secreted by prostate gland epithelia. This leads toward further promotion of immunologic response inducing chronic inflammation. From other tumor models it has been known that long lasting chronic inflammation stimulates cellular proliferation and favors fasten growing, which is often in hypoxic circumstances deranged at certain level. Oxygen delivery and consumption are regulated with hypoxia-inducible factors (HIFs) among other molecules, which also induce transcription of VEGF stimulating, in turn, tumor angiogenesis due to increased oxygen demand. Hypoxic cancer cells are stimulated to acquire invasive and metastatic properties and they are promptly developing resistance to chemotherapy too (11). Although significance of inflammation in cancer progression has been discussed and researched for a long time, till recently, research has extrapolated pathways directed to connection between inflammation and cancer evolution. Different studies strongly point out that chronic inflammation is involved in progression of chronic prostatic disease, such as benign prostatic hyperplasia (BPH) and consequent development of prostate cancer (12). The coincidence of chronic inflammation and tumorigenesis in the peripheral zone identified as so-called proliferative inflammatory atrophy is proposed as possible precursor of prostatic intraepithelial neoplasm. This can be explained since inflammatory microenvironment releases growth factors and cytokines that may influence the activation of the vascular endothelial cells and signal transduction in these cells. Continuous inflammatory stimuli as it have been previously shown result in endothelial sprouting. This links process of inflammation with angiogenesis, which predisposes preexisting endothelial cells to continuous activation and usually has its gene activation for consequence. Neovascularization includes also presence of dysfunctional endothelial cells, which is particularly present during chronic inflammation and among others related with sustained p38 MAP kinase pathway activity and increased levels of migration of cytokines. When observed, described gene changes reactive oxygen species (ROS) levels are elevated (13). The novel factors could possibly influence angiogenic switch consequently leading to progression from low-grade prostatic intraepithelial neoplasia (PIN) to high-grade PIN and beyond to prostate cancer or even more aggressive, poorly differentiated, and androgen-independent histological subtypes.

From morphological point of view, it is important to recognize the presence of even small amount of microscopic patterns, which may be responsible for prompt tumor progression, advancement, and transition from low-grade lesion to the high grade one.

Today, we are aware of their importance and connection with different molecular pathways, which prevail in interplay among tumor cells and environment interaction. Recently, it has been shown that the p38-MAPK pathway can be activated under continuous extensive anti-androgen exposure in prostatic cancer cells, and that the p38-MAPK pathway has a critical role in the induction of resistance, as well as in the acquisition of a more aggressive and invasive phenotype (13). This observation is at least interesting if we would not exaggerate since it offers potentially new therapeutic escape window. Namely, proposed role of p38 in controlling the phosphorylation and activity of transcription factors that has been established would regulate tumor and stromal cells proliferation and the expression and release of multiple tumor-derived cytokines. Cytokines may in turn promote tumor angiogenesis and lymphangiogenesis, thereby favoring the establishment of a permissive tumor microenvironment. The prostate microenvironment appears relatively simple in comparison with some other milieus (i.e., bone marrow for instance) since it consists of the components of supporting stromal elements’ extracellular matrices, and predominantly, carcinoma-associated fibroblasts. Activation and increased expression of several adhesion molecules correlate with a progression of malignant tumors. CD44 has been recognized to recruit and accumulate matrix metalloproteinase on the cell surface, enabling indirectly tumor cell angiogenesis and invasion (14). CD44 is also recognized as a potential target for cells gained pre-metastatic phenotype and entering process of epithelial–mesenchimal transition (15). Understanding a different expression of CD44s marks and metalloproteinase in the field of invasion may help in recognizing patients with more aggressive forms of prostate tumors, who should be treated surgically and also additionally. Fibroblast actively participates in extracellular matrix remodeling thereby promoting actively carcinogenesis and stimulating prostate cancer cell proliferation and angiogenesis (15, 16). From this concept, two options of therapeutic actions are deriving one targeting reactive stroma and other is aiming inflammatory, stromal and circulating cells (17). There is a constant need of new anticancer agents, in general oncologic therapy. Though exact molecular events enabling prostate cell malignant transformation and the ability to gain aggressive metastatic phenotype remain elusive, it is important not to overlook the importance that new therapeutic agents should be able to provide a therapeutic solution for both local and metastatic prostate cancer. As previously mentioned, as agents capable of disrupting MAPK signaling pathway and inducing irreversible inhibition of tumor proliferation, angiogenesis and metastatic potential, and as moderators of expression and function of adhesion molecules, molecule CD44 would probably be the next generation of promising therapeutics for management of prostate cancer.

## Conflict of Interest Statement

The authors declare that the research was conducted in the absence of any commercial or financial relationships that could be construed as a potential conflict of interest.
